# A process evaluation of PRONTO simulation training for obstetric and neonatal emergency response teams in Guatemala

**DOI:** 10.1186/s12909-015-0401-7

**Published:** 2015-07-24

**Authors:** Dilys M. Walker, Francesca Holme, Sarah T. Zelek, Marisela Olvera-García, Airaín Montoya-Rodríguez, Jimena Fritz, Jenifer Fahey, Héctor Lamadrid-Figueroa, Susanna Cohen, Edgar Kestler

**Affiliations:** 1 Department of Obstetrics and Gynecology, University of Washington, Seattle, WA 98104 USA; 2 Department of Global Health, University of Washington, 325 9th Ave, Box 359931, Seattle, WA 98104 USA; 3 Division of Reproductive Health, Research Center for Population Health, National Institute of Public Health, Universidad No 655, Col Santa Maria Ahuacatitlan, CP 62100, Cuernavaca, Morelos Mexico; 4 Department of Obstetrics and Gynecology, University of Maryland School of Medicine, 22 S Green St, Baltimore, MD 12201 USA; 5 College of Nursing, University of Utah, 10 South 2000 East Salt, Lake City, UT 84112 USA; 6 Epidemiological Research Center in Sexual and Reproductive Health (CIESAR), Guatemala City, Guatemala

**Keywords:** In-situ simulation, Continuing medical education, Emergency obstetric care, Inter-professional training

## Abstract

**Background:**

Despite expanding access to institutional birth in Guatemala, maternal mortality remains largely unchanged over the last ten years. Enhancing the quality of emergency obstetric and neonatal care is one important strategy to decrease mortality. An innovative, low-tech, simulation-based team training program (PRONTO) aims to optimize care provided during obstetric and neonatal emergencies in low-resource settings.

**Methods:**

We conducted PRONTO simulation training between July 2012 and December 2012 in 15 clinics in Alta Verapaz, Huehuetenango, San Marcos, and Quiche, Guatemala. These clinics received PRONTO as part of a larger pair-matched cluster randomized trial of a comprehensive intervention package. Training participants were obstetric and neonatal care providers that completed pre- and post- training assessments for the two PRONTO training modules, which evaluated knowledge of evidence-based practice and self-efficacy in obstetric and neonatal topics. Part of the training included a session for trained teams to establish strategic goals to improve clinical practice. We utilized a pre/post-test design to evaluate the impact of the course on both knowledge and self-efficacy with longitudinal fixed effects linear regression with robust standard errors. Pearson correlation coefficients were used to assess the correlation between knowledge and self-efficacy. Poisson regression was used to assess the association between the number of goals achieved and knowledge, self-efficacy, and identified facility-level factors.

**Results:**

Knowledge and self-efficacy scores improved significantly in all areas of teaching. Scores were correlated for all topics overall at training completion. More than 60 % of goals set to improve clinic functioning and emergency care were achieved. No predictors of goal achievement were identified.

**Conclusions:**

PRONTO training is effective at improving provider knowledge and self-efficacy in training areas. Further research is needed to evaluate the impact of the training on provider use of evidence-based practices and on maternal and neonatal health outcomes.

**Trial registration:**

NCT01653626

## Background

Access to a skilled birth attendant and quality emergency obstetric care are important factors that improve maternal and perinatal mortality [1–3]. Although the majority of maternal and perinatal deaths could be prevented with appropriate emergency obstetric care, not all women receive medical attention when needed [4–10]. There are many reasons why women do not have timely access to quality services, including the “three delays”: the delay in decision to seek care; delay in reaching appropriate care in time; and/or the delay in delivery of appropriate care once at a facility [4, 11].

In Guatemala, where neonatal mortality in 2012 was reported as 15 per 1000 live births and the 2010 adjusted maternal mortality was 120 deaths per 100,000 live births, only 51.5 % of women deliver with skilled care providers [12]. This percentage is even lower in indigenous, impoverished areas [13]. To address this problem, the Guatemalan government, with World Bank funding, opened clinics with 24-h labor and delivery care known as C*entros de Atención Permanente* (CAPs) throughout the country [14]. However, despite these infrastructural developments, mortality remains largely unchanged over the last decade [15]. If sustainable improvements are to be achieved in Guatemala, the complex nature of this problem demands an interdisciplinary, multilevel approach.

A multi-pronged intervention package was designed and implemented in northern Guatemala to address low utilization of clinic-based services, weak community-level linkages between traditional birth attendants (TBAs) and the formal health system, and inadequate provision of emergency obstetric care. The three intervention elements included: 1) community social marketing campaign promoting institutional delivery; 2) professional midwife liaisons working with TBAs; and 3) PRONTO^SM^ (*Programa de Rescate Obstétrico y Neonatal: Tratamiento Óptimo y Oportuno*), a highly realistic simulation-based obstetric and neonatal emergency and team training program. The goal of this novel and unique training was to improve the quality of care provided in the facilities, and ultimately, perinatal mortality.

Traditional didactic approaches have been found to be of limited effectiveness in improving the management of obstetric emergencies [16]. Simulation-based training has emerged as a key training modality for providers of emergency obstetric care as evidence shows it improves clinical practice, communication, and teamwork skills [17–19]. However, the majority of simulation training is resource-intensive, making its translation into limited-resource settings challenging [20, 21]. PRONTO was developed in Mexico in 2009 as an innovative low-cost, highly realistic, medical training solution for obstetric and neonatal interprofessional care teams [22], and it is thought that this novel program could improve practice in Guatemala, where no such training exists. PRONTO is the only in-situ, highly realistic simulation and team-training program for limited resource settings of its kind. This paper reports on the impact of the PRONTO on provider knowledge, self-efficacy, and clinic goal achievement. We hypothesize that PRONTO improves healthcare provider knowledge and self-efficacy and promotes achievement of team goals, important practice components that can improve care quality and outcomes in Guatemalan clinics.

## Methods

### Trial design and participants

The PRONTO training intervention was carried out between July and December 2012 at 15 intervention facilities in the four Northern Guatemalan districts of San Marcos, Alta Verapaz, Quiche, and Huehuetenango. These clinics received PRONTO training as part of a larger, pair-matched, cluster randomized controlled trial in 30 clinics with clinical training and community components. This report is limited to an analysis based on pre/post tests administered only to intervention clinics, as control clinics received no intervention and provider testing was not feasible at those sites.

Clinics were excluded from the study if they were not open 24-h, had an operating room, had fewer than an average of seven births per month (in the six months prior to the intervention), or if they were located more than six hours by vehicle to the district’s commercial center. All clinics were located in communities with TBAs and had basic equipment and personnel necessary for vaginal deliveries. In Guatemala, TBAs have no formal training and in recent years the government has encouraged incorporating TBAs into clinics as support staff and doulas. The PRONTO team encouraged clinics to invite community TBAs to participate in the training. Training participants were chosen by clinic directors from the medical personnel who care for women and babies during labor and birth or immediately postpartum and their roles in the simulations were based on their real scope of practice. Providers at the intervention clinics voluntarily participated in the training and received no compensation other than their typical salary, transportation and meals. All trainees were informed that they were in the intervention arm of the trial, and provided verbal informed consent to the use of their written evaluations for research purposes. The study was reviewed and approved by the World Health Organization (WHO) Research and Ethics Review committee, the Independent Latin Ethics Committee, the Guatemalan National Committee for Ethics in Health, Ministry of Public Health, and the University of Washington IRB, Seattle (Application # 41922). The trial is registered at the NIH Current Cluster Trial Registration database (http://www.clinicaltrials.gov; identifier: NCT01653626). The study setting and methods were published elsewhere [23].

### Intervention

The PRONTO training intervention utilizes highly realistic yet low-technology, low-cost simulation materials. Simulation scenarios take place within the actual clinic setting using only resources that are normally available on-site. PartoPants™, the hybrid simulator worn by a patient actress to simulate obstetric emergencies, were designed by the PRONTO team [24]. PartoPants™ are made from recycled surgical scrub pants and are modified to have a vaginal opening and pocket to hold an IV bag with tubing for simulated blood. A cloth doll and placenta, delivered through the PartoPants™, are used to simulate the neonate and placenta during birth. The Laerdal NeoNatalie© is used for neonatal resuscitation scenarios.

**Fig. 1:**
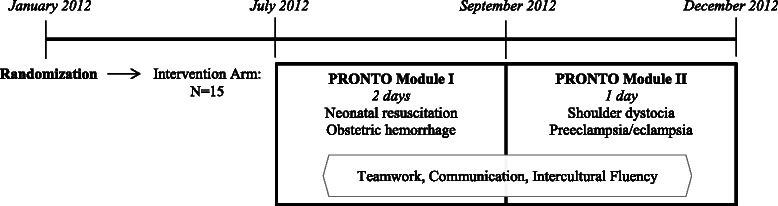
Timeline and components of PRONTO training intervention in Guatemala

PRONTO consists of two modules conducted two to three months apart that cover topics of teamwork, communication, intercultural fluency, obstetric hemorrhage, neonatal resuscitation, preeclampsia/eclampsia, and shoulder dystocia (Fig. 1). Training sessions have minimal formal didactics and are comprised of interactive team-building exercises, case-based learning, targeted skill sessions, simulation of obstetric and neonatal emergencies, video-guided debriefings and strategic planning sessions. Teamwork and communication concepts, adapted from Team STEPPS™ Training Program, are taught and reinforced throughout both modules [25, 26]. Evidence-based practices for physiologic birth and respectful, culturally humble care are also integral components. The curriculum is based upon WHO standards in maternal and newborn care, and was modified for use in Guatemala to meet the Guatemalan Ministry of Health guidelines [27]. The training was specifically adapted to address common cultural and language barriers between the patient population and the facility providers in Northern Guatemala by addressing cultural humility and humanized birth training in the modules [28]. Finally, during the strategic planning sessions, the provider teams identify actions aiming for clinical improvement based on weaknesses, they self-identify through the simulation and debriefs. No intervention took place at the control clinics.

### Outcome measures

The primary outcomes included average change in participant self-efficacy and knowledge scores for each training topic area. Training participants at each intervention clinic completed pre- and post- training questionnaires for Modules I and II, which evaluated knowledge of evidence-based practice and confidence in their own ability to perform key skills (self-efficacy). The questionnaires were adapted from instruments used in the PRONTO pilot and subsequent hospital-level controlled trial in Mexico. The Module I pretest and posttest included 58 questions, which assessed knowledge and self-efficacy of obstetric hemorrhage and neonatal resuscitation, and general obstetric emergency self-efficacy. The Module II pretest consisted of 36 questions to assess knowledge and self-efficacy on topics of preeclampsia/eclampsia and shoulder dystocia. The Module II post-test included 94 topical questions from all of the five topics covered throughout Module I and II and was administered at the end of the training intervention. Questionnaires were anonymous and participants were given a study ID to link the pre-post module answers. Knowledge for each topic area was assessed on a scale of 0–100 based on the percentage of correct questions answered. Answers left blank or incorrectly marked with multiple responses were coded as wrong. For each self-efficacy question, participants were asked to assess their level of confidence in performing an array of simple to complex practices related to obstetric and neonatal emergencies using scales of 0–100 based on Bandura’s model of self-efficacy [29, 30]. Scores for each topic area were averaged based on these scales.

Trainees worked in clinic-based teams to decide on goals for improving obstetric/neonatal care, teamwork, work processes, and infrastructure at their site at the end of Module I during a facilitated quality improvement planning session. We grouped goals into four broad categories (teamwork, training, healthcare systems change, and intercultural fluency). There was no stipulation on the number of goals clinics were asked to select. During a follow-up session at the beginning of Module II, trainers evaluated their teams’ achievement of set goals (yes/no). Goal achievement was self-reported, but when possible, system change goals were confirmed by direct observation by PRONTO training personnel. Ongoing research is underway to examine the extent of this intervention’s impact on practice improvement. However, for this phase of the study, clinical improvement goal achievement was used as a proxy measure of practice change.

### Statistical analysis

We used descriptive statistics to characterize participating clinics and trainees. Knowledge and self-efficacy changes were assessed in the areas of obstetric hemorrhage, neonatal resuscitation, preeclampsia/eclampsia, and shoulder dystocia. Change in general obstetric emergency self-efficacy was also assessed. Variables for overall changes in knowledge/self-efficacy for Module I and for Module II were also created. A combined variable for overall knowledge change from the start of the training Module I until the conclusion of training Module II was created for obstetric hemorrhage and neonatal resuscitation. We estimated the impact of the training on test scores by fitting a longitudinal fixed-effects linear regression model with fixed effects at the individual level. The outcome variable was the test score of knowledge or self-efficacy and the dummy variables for time (pre/post training) served as independent variable. The model is represented by the equation:

$$ {Y}_{i,t}-{Y}_{i,0}={\beta}_1{t}_1+{\beta}_2{t}_2+\left({\varepsilon}_{i,t}-{\varepsilon}_{i,0}\right) $$

Where *Y*_*i*,*t*_ is the score for individual i at time t and *Y*_*i*,0_ is the score at baseline, *t*_1_ *and t*_2_are dummy variables for the first and second measurements and *β*_1_, *β*_2_ are the average differences in score relative to baseline, *ε*_*i*,*t*_ represents random variability at time t. The fixed-effects approach yields an estimate of the within-subject change in the outcome variables (knowledge and self-efficacy) [31]. Robust standard errors with clustering were calculated to take into account within-facility correlation [32]. A second set of models included a time*facility interaction term to test for possible between-facility heterogeneous effects of the training. To characterize the relationship between knowledge and self-efficacy score, the correlation between the two scores was calculated using Pearson’s correlation coefficient for both baseline and follow-up values. Finally, we used descriptive statistics to describe goal setting and achievement.

As a secondary exploratory analysis, we analyzed clinic-level factors possibly associated with goal achievement. To do this, we used Poisson regression with number of goals achieved as the response variable, controlling for number of goals set. Covariates tested for an association with total number of goals achieved were pre/post Module I average change in self-efficacy, pre/post Module I average change in knowledge, training site equipment availability, percent of overall staff attending training, and average age, sex, and profession of participating providers. These variables were selected for the plausibility of an association with success in achieving established goals.

All results are presented as overall and stratified by profession given a previous and reasonable learning differential for auxiliary nurses versus doctors/professional nurses. Significance was defined at p-value < 0.05, and all analyses were performed using STATA v. 12.0 [33].

## Results

We conducted seven complete trainings. Eight of the clinic teams attended training off-site at neighboring clinics. Overall, 219 providers from 15 clinics participated in the PRONTO training intervention, with on average 28 participants at each of the training sessions. Our analysis is limited to the participants (*n* = 207) who completed the questionnaires for Module I and/or Module II. Questionnaires were designed for doctors and nurses; however, one of the nine TBAs completed a questionnaire, which was included in the analysis. Clinics were on average 51.2 km away from the most used referral hospital and were staffed with 8.6 physicians and professional nurses and 13.5 auxiliary nurses (Table 1). An average 63.0 % of eligible providers were represented in the trainings.

**Table 1:** PRONTO training facility and participant characteristics at intervention hospitals

Characteristics	Mean (SD)^a^	Min	Max
Facilities (*N* = 15)			
Distance (Km) to most referred to hospital			
*San Marcos*	17.0 (16.7)	2.0	35.0
*Huehuetenango*	83.3 (43.8)	33.0	113.0
*El Quiche*	38.7 (17.2)	18.0	60.0
*Alta Verapaz*	62.4 (60.6)	8.0	136.0
*Total*	51.2 (44.6)	2.0	136.0
Total personnel staffing			
*Physicians & professional nurses*	8.6 (2.6)	5.0	14.0
*Auxiliary nurses*	13.5 (4.6)	7.0	24.0
Percentage of personnel trained by PRONTO			
*San Marcos*	57.2 (15.9)	38.5	60.0
*Huehuetenango*	76.2 (21.1)	60.0	100.0
*El Quiche*	64.9 (33.2)	23.8	95.0
*Alta Verapaz*	56.9 (14.5)	43.5	76.5
*Total*	63.0 (21.3)	23.8	100.0
Participating Personnel (*N* = 207)^b^			
Age (years)	34.9 (9.6)	20	67
Sex, N (%)		-	-
*Females*	126 (62.1)		
*Males*	77 (37.9)		
Provider type, N (%)		-	-
*Auxiliary nurses*	121 (59.6)		
*Physicians & professional nurses*	80 (39.4)		
*Other*	2 (1.0)		

^a^Mean (SD) unless otherwise noted. ^b^Completed at least one pre- or post- questionnaire. Missing values for age *n* = 10, sex *n* = 4, and provider type *n* = 4

Knowledge in all subject areas improved significantly (p < 0.001) following the training intervention (Table 2). Self-efficacy changes were similar to the knowledge improvements and were all significant at <0.001 (Table 2). A joint test of the time*facility interaction terms yielded no evidence of effect heterogeneity between facilities, except for the obstetric hemorrhage knowledge score, however, even in this case, only two clinics showed a significantly larger effect in the first follow-up evaluation (San Pedro and Tejutla) p < 0.01. For both knowledge and self-efficacy, improvements were similar for all personnel types, however, doctors and professional nurses had higher pre- and post- scores in all topics compared to auxiliary nurses. Knowledge and self-efficacy were found to be positively correlated for all topics at training completion (Table 3) as trainees with high self-efficacy were also found to have high knowledge scores. When stratified by profession, the correlation remained significant for auxiliary nurses only in obstetric hemorrhage and preeclampsia/eclampsia. Doctors and professional nurses, however, had no correlation between knowledge and self-efficacy topics except for shoulder dystocia post-training.

**Table 2:** Change in knowledge and self-efficacy scores by profession for management of obstetric and neonatal emergencies^a^

Variables	*All*			*Doctors/Professional Nurses*			*Auxiliary Nurses*		
	Pre Score^b^	Post I Change	Post II Change	Pre Score	Post I Change	Post II Change	Pre Score	Post I Change	Post II Change
Knowledge									
Obstetric hemorrhage	27.5	11.5	9.5	31.9	15.2	11.5	24.5	9.0	9.1
Neonatal resuscitation	34.8	23.4	16.3	42.4	25.7	19.3	29.4	22.3	16.3
Combined Module I	30.4	19.0	14.2	36.6	21.4	16.5	26.3	17.6	12.8
Preeclampsia/Eclampsia	41.5	--	13.5	47.2	--	15.9	38.2	--	12.7
Shoulder dystocia	48.6	--	13.9	60.5	--	11.5	42.3	--	15.6
Combined Module II	45.0	--	13.7	53.9	--	13.7	40.2	--	14.1
Self-efficacy									
General emergencies	83.9	7.5	8.8	87.2	8.0	8.5	81.8	7.2	8.8
Obstetric hemorrhage	79.5	8.6	8.6	82.7	10.9	10.1	77.2	7.7	8.1
Neonatal resuscitation	74.8	16.0	15.6	78.6	16.4	15.7	72.3	16.2	15.6
Combined Module I	78.6	11.5	11.6	82.9	11.5	11.3	75.8	11.7	11.8
Preeclampsia/Eclampsia	72.7	--	14.0	77.8	--	14.2	69.9	--	14.5
Shoulder dystocia	59.1	--	28.3	65.4	--	26.4	55.0	--	30.5
Combined Module II	65.7	--	21.4	71.2	--	20.6	62.4	--	22.6

^a^*N* = 193 for pre/post I, *N* = 161 for pre/post II and *N* = 159 for pre I/post II. All changes significant with p-value <0.001. Topics not taught and tested as part of module I are marked blank with “--”. ^b^Pre-score from Module I for obstetric hemorrhage, neonatal resuscitation, and general emergencies, Pre-score from Module II for Preeclampsia/eclampsia and shoulder dystocia. Knowledge scores are percent correct and self-efficacy scores are self-score out of 100

**Table 3:** Correlation between knowledge and self-efficacy by topic and stratified by profession using Pearson’s correlation coefficients

Training Topics	Pre		Post Module I		Post Module II	
	r	p-value	r	p-value	r	p-value
Overall, *N* = 207						
Obstetric hemorrhage	0.27	<0.001	0.31	<0.001	0.27	<0.001
Neonatal resuscitation	0.19	0.01	0.14	0.05	0.25	<0.001
Preeclampsia/ Eclampsia	0.13	0.11	--	--	0.26	<0.001
Shoulder dystocia	0.24	<0.001	--	--	0.30	<0.001
Auxiliary Nurse, *N* = 121						
Obstetric hemorrhage	0.40	<0.001	0.32	0.01	0.40	<0.001
Neonatal resuscitation	0.12	0.32	0.14	0.22	0.21	0.13
Preeclampsia/ Eclampsia	0.04	0.78	--	--	0.29	0.03
Shoulder dystocia	0.11	0.43	--	--	0.22	0.10
Doctors & Professional Nurses, *N* = 80						
Obstetric hemorrhage	0.13	0.19	0.15	0.15	0.10	0.32
Neonatal resuscitation	0.16	0.12	0.01	0.89	0.14	0.18
Preeclampsia/ Eclampsia	0.11	0.26	--	--	0.11	0.25
Shoulder dystocia	0.23	0.02	--	--	0.23	0.02

Overall, a total of 93 goals were identified following Module I strategic planning sessions. Types and examples of goals are described in Table 4. On average, each clinic set 5.5 goals and overall 66 % of all goals set were considered achieved at follow-up. Knowledge change, self-efficacy change, average participant age, proportion of males participating, proportion of all clinic staff attending the training, proportion of physicians and professional nurses attending, and the equipment index (composite indicator of the clinic’s supplies, equipment, and medications) were tested as possible indicators of the number of goals achieved. However, no potential predictors of goal achievement were statistically significant in either the crude or multivariate Poisson analysis.

**Table 4:** Examples of strategic goals achieved and unachieved by category

Goal Category:	Teamwork & Communication	System & Infrastructure	Intercultural Fluency	Training
Goals Identified:	15	42	17	19
Goals Achieved:	66.7 %	69.8 %	70.6 %	55.3 %
		*Examples of Achieved Goals*		
	Assign leadership roles for obstetric emergencies	Reorganize/rearrange the clinic	Invite traditional midwives to deliveries to improve patient care	Train the rest of the clinic in topics/skills learned in the PRONTO training with PartoPants
	Implement SBAR and other communication techniques	Request more personnel and support for infrastructure improvements	Ask pregnant women questions (i.e., in what position would you like to delivery)	Promote PRONTO trainings
	Set-up meetings with personnel to improve communication and organization	Create a roster for high risk pregnancies	Develop a traditional delivery space	Continue training staff on high risk pregnancies
	Use and disseminate the communication rules	Replace neonatal resuscitation table	Allow drinking of hot water during labor	Train the traditional midwives using simulation.
		*Examples of Unachieved Goals*		
	Include those not attending a delivery in the management of emergency situations	Request a bed and other equipment to offer vertical births.	Ask women which position she prefers to deliver in	Continued training of staff on obstetric emergencies
	Implement and replicate the rules of communication learned	Conduct two supply inventories	Try offering vertical deliveries	Replicate PRONTO with the rest of the staff
	Remind the group to improve communication	Streamline drug procurement processes	-	Sensitize teams to lessons learned

## Discussion

PRONTO training improved knowledge and self-efficacy training topic scores and promoted achievement of goals designed for clinical improvement. The overall rate of goal achievement suggests that simulation training may promote team-based quality improvement and practice change. PRONTO training was considered “very important” for their clinic by 100 % of evaluation respondents, and 97 % (*N* = 181) of the responding providers (*N* = 186) said they would use the learning and teamwork concepts in their future work.

Previous evaluations of similar process indicators following PRONTO training in Mexico found comparable results [22, 34]. Other simulation-based training courses have also found that their courses improved provider knowledge, teamwork, communication, and neonatal or maternal outcomes [20, 35–41]. PRONTO, however, is the first course to integrate team training with simulation and care of the maternal-neonatal dyad in low-resource settings.

Several study limitations warrant discussion. While the clinics were randomly selected, individual participation was not random and data was missing from participants who did not complete a questionnaire. Thus there is the potential for selection bias. It was not feasible to administer questionnaires in control clinics due to logistical and resource constraints. Thus, as with any non-experimental pre/post study design, we are unable to account for potential historical, maturation, testing bias that could be responsible for some changes observed. Although the correct answers to the questionnaires were never provided explicitly, we cannot discount potential testing bias as the same test was administered as the pre and post- test. Also, it is likely that there is potential for self-reporting bias, particularly with regard to goal achievement. We did our best to limit this by verifying goals achieved in person whenever possible. We acknowledge that these results showing improved test scores does not necessarily translate into actual practice improvement as that cognitive knowledge does not necessarily translate into clinical ability.

While the questionnaire was utilized in two previous studies, it has not been officially validated due to limited time and resources. However, there has been consistency in the results obtained in varied settings (Mexico, Guatemala, Kenya). One recurrent mistake made by participants was selection of multiple answers when the answer specified to “select one”. This problem was more common on the post-test when participants are usually eager to leave and so, perhaps, reflects the rush of the test-taker. These multiple selected answers were coded as “wrong” for our analysis, which makes our results conservative. At completion of this study, the questionnaire was edited to ensure future clarity.

## Conclusions

Our findings suggest that PRONTO training effectively improves provider knowledge and self-efficacy of evidenced-based practice in training topic areas, and promotes goal achievement. PRONTO is the only emergency obstetric and neonatal training, we are aware of, that combines in-situ simulation with team training, with evidence to support improvements in knowledge and self-efficacy among providers. Globally, with widespread recommendation for obstetric and neonatal simulation-based training, the feasibility of adapting PRONTO for implementation in additional low- and high resource settings should be assessed. Following the intervention, supported by our significant results and overwhelmingly positive response by participants and authorities, additional funding was sought and obtained to expand roll-out of the intervention packet in Huehuetenango and Alta Verapaz beyond the intervention facilities, which is now underway. There is ongoing interest in further roll-out in San Marcos and Quiche. More research is needed on the impact of these positive changes on actual maternal and perinatal morbidity and mortality measures, and our impact data is currently being analyzed.

## Abbreviations

PRONTO: *Programa de Rescate Obstétrico y Neonatal: Tratamiento Óptimo y Oportuno* (Obstetric and Neonatal Emergency Training for Optimal and Timely Treatment)
TBA: Traditional birth attendant
WHO: World Health Organization
